# Identification of gain- and loss-framed cancer screening messages that appeared in municipal newsletters in Japan

**DOI:** 10.1186/1756-0500-7-896

**Published:** 2014-12-11

**Authors:** Tsuyoshi Okuhara, Hirono Ishikawa, Hiroko Okada, Takahiro Kiuchi

**Affiliations:** Department of Social Medicine, Graduate School of Medicine, The University of Tokyo, 7-3-1 Hongo, Bunkyo-ku, Tokyo, 113-8655 Japan; Department of Health Communication, School of Public Health, The University of Tokyo, 7-3-1 Hongo, Bunkyo-ku, Tokyo, 113-8655 Japan

**Keywords:** Message framing, Cancer screening, Municipal newsletter, Persuasion

## Abstract

**Background:**

Research suggests that cancer screening messages are more persuasive when framed in terms of the costs of not obtaining screening (i.e., loss-framed) than when framed in terms of the benefits of obtaining screening (i.e., gain-framed). However, to what extent these findings have been integrated into public health practice is unknown. To analyze message framing of cancer screening information, the present study examined message framing of cancer screening announcement articles that appeared in municipal newsletters published from 23 wards in central Tokyo, Japan. Two independent raters coded the articles. Gain- and loss-framed sentences in each article were identified, and based on what the sentences conveyed, articles were classified into gain-framed, loss-framed, mixed-framed, and non-framed.

**Result:**

Inter-rater reliability was acceptable (intraclass correlation coefficient = 0.88). Of the 129 articles evaluated, the total number of gain-framed sentences was 87, while that of loss-framed sentences was six. The total number of gain-framed articles was 32 (24.8%) while that of loss-framed articles was zero (0%). Five (3.9%) articles were mixed-framed. Ninety-two (71.3%) articles were non-framed.

**Conclusions:**

Cancer screening announcement articles of municipal newsletters were mostly non-framed or gain-framed in 23 Tokyo wards in Japan. The absence of loss-framed articles and only a small number of loss-framed messages indicate a missed opportunity to persuade readers to obtain cancer screenings. Loss-framed messages and articles need to be increased to enhance the persuasiveness of cancer screening information in municipal newsletters.

## Background

Cancer is the leading cause of death in many developed countries, including Japan. In Japan, 805,236 new cases of cancer and 360,963 deaths from cancer were estimated in 2010 and 2012 [1]. Municipalities have been responsible for providing cancer screenings since population-based screening for cancer was introduced under the Health and Medical Service Act for elderly people in 1983. However, cancer screening rates in Japan are lower than those in Western countries and Korea. The screening rates for breast cancer (percentage of females aged 50–69 screened) and cervical cancer (percentage of females aged 20–69 screened) in 2010 were 80.4% and 85.0% in the United States, 77.0% and 78.6% in the United Kingdom, and 63.6% and 63.8% in Korea, whereas they were 36.4% and 37.7% in Japan, respectively [2]. Other cancer screening rates in Japan (percentage of males and females aged 40–69 screened) are similarly low as follows: gastric cancer is 32.3%, lung cancer is 24.7%, and colorectal cancer is 26.0% [3].

There are multifactorial barriers to cancer screening, such as knowledge, attitudes and beliefs of patients and providers, socioeconomic factors of patients, the medical system (e.g., accessibility of screening test, lack of tracking and follow-up care, cost of screening test) [4]. A multimodality approach is required to overcome these barriers and improve the screening rates. In particular, effective communication of cancer and cancer screening information is of paramount importance [5]. Providing relevant information alone is widely acknowledged as not being sufficient to motivate people to adopt a healthy behavior. Persuasion is inherent in the efforts of health promotion and manifold persuasive communication strategies have been explored [6]. One of those strategies is the framing postulate of Prospect Theory [7].

Prospect theory purports that people tend to take risks when they evaluate options in terms of associated costs but to avoid risks when the same options are described in terms of associated benefits [8]. Hence, when the message of the choice is framed to highlight potential losses, people are more willing to choose risky options to prevent those losses. Conversely, when the choice is framed to highlight potential gains, people are less willing to choose risky options to secure those gains. Health messages can be framed to emphasize either the benefit of adopting a particular behavior (i.e., gain-framed message) or the negative consequence of not adopting a particular behavior (i.e., loss-framed message). Previous research has suggested that how the health message is framed can alter the persuasive impact of the message [7–10].

Rothman and Salovey [7] applied the framing postulate of Prospect Theory to the health promotion domain and proposed that (i) loss-framed messages should be more effective for promoting risky health behavior, such as detection of illness (e.g., obtaining mammography screening involves a higher degree of risk that a serious illness could be discovered) and (ii) gain-framed messages should be more effective for promoting minimally risky health behavior, such as preventive behavior (e.g., increasing physical activity involves little risk). According to this hypothesis, cancer screening messages should be more persuasive when loss-framed, focusing on the negative consequence of not being screened.

A growing body of evidence supports this hypothesis. A previous study showed that women who read a loss-framed pamphlet that emphasized the risk of not performing a breast self-examination (BSE) showed more positive attitudes and intentions toward BSE and practiced BSE more frequently than women who received a gain-framed or non-framed pamphlet, or no information [11]. Similarly, women who are exposed to loss-framed messages are six times more likely to obtain a mammogram than those who receive the usual message [12]. Further, loss-framed messages are reported to be more effective in promoting prostate cancer screening [13] and skin cancer screening [14].

The fear appeal (persuasive message that arouse fear) is also one of the persuasive strategies that has been effectively used in health promotion campaigns [15, 16]. Hale and Dillard suggest that fear appeals should be loss-framed to emphasize negative consequences for not following message recommendations [16]. Thus, loss-framed messages are also considered to be useful to utilize the fear appeal.

Although the effectiveness of message framing for health promotion has been reported repeatedly for almost 2 decades, to what extent this finding is used in practice to communicate cancer screening information is unknown. In Japan, most of the municipalities publish municipal newsletters for the residents to disseminate administrative services including health services such as cancer screenings [17]. In fact, municipal newsletters are most frequently mentioned by residents as a source of cancer screening information [18]. Therefore, improving cancer screening announcements that appear in municipal newsletters is important for effective communication of cancer screening information in Japan. This study aimed to analyze message framing of cancer screening announcement articles that appeared in municipal newsletters published in Tokyo, Japan. The secondary aim of this study was to discuss how to improve cancer screening announcements by applying the framing postulate of Prospect Theory.

## Methods

Articles including cancer screening announcements were collected from municipal newsletters that were published in central Tokyo (23 wards) from January to December 2013. Each of the 23 wards publishes newsletters for the residents twice to four times a month. The volume of the newsletters is from four to 16 pages of A3 paper size. We downloaded the PDF data of the municipal newsletters from the website of each ward in April 2014 to use in analyses. During the sampling period, 257 articles including cancer screening announcements were identified. Because some of the articles were posted more than twice, the number of unique articles was 129.

The presence of gain- or loss-framed messages was investigated in the 129 unique articles. Message coding guidelines were created based upon previous studies [7, 19, 20] (Table 1). Initially, the first author and one of the co-authors conducted a preliminary analysis by applying the coding guidelines to three randomly selected articles to resolve any discrepancies in interpretation. The first author then coded each sentence of all of the 129 articles and identified gain- and loss-framed sentences. The number, mean value, maximum, and minimum of gain- and loss-framed sentences were described. According to the type and presence of framed sentences that were conveyed, articles were classified into four categories: gain-framed articles (i.e., conveying gain-framed and non-framed sentences), loss-framed articles (i.e., conveying loss-framed and non-framed sentences), mixed-framed articles (i.e., conveying gain-framed, loss-framed, and non-framed sentences), and non-framed articles (i.e., conveying only non-framed sentences, and no gain- or loss-framed sentences). The number and percentage of each category were calculated. To examine the inter-coder reliability, previous studies typically assessed 10-20% of the total sample by two independent coders [21–24]. In general, assessing not all but a part of the total sample is beneficial to researchers for making easier to reproduce the study. Hence, we chose the longest article from each of the 23 wards (18% of the total sample). Then, one of the co-authors independently coded the 23 articles to examine the inter-coder reliability.

**Table 1:** **Message coding guidelines and sample messages**

Coding guideline		Example sentence
Message frame	Emphasis of messages	
Gain-framed	(a) The benefits of cancer screening.	When you obtain cancer screenings, you are taking advantage of the best method for detecting cancer early.
		The advantage of detecting cancer early is that you are more likely to increase your treatment options.
		Detecting cancer early can save your life.
	(b) The costs avoided by cancer screening.	If a cancer is detected early, it is less likely to be fatal.
		The advantage of detecting cancer early is that you may need less radical procedures.
		Detecting cancer early can reduce your medical costs.
Loss-framed	(a) The costs of not obtaining cancer screening.	Failing to detect cancer early can cost you your life.
		The disadvantage of failing to detect cancer early is that you may need radical procedures.
		If cancer is detected late, it is more likely to be fatal.
	(b) The benefits missed by not obtaining cancer screening.	When you avoid obtaining cancer screening, you are failing to take advantage of the best method for detecting cancer early.
		The disadvantage of failing to detect cancer early is that you may have fewer treatment options.

Data were analyzed using the Statistical Package for the Social Sciences version 21.0 (SPSS, Chicago, IL, USA). Inter-rater reliability was assessed using the intraclass correlation coefficient (ICC) because the numbers of gain- and loss-framed sentences of each article were described by two coders and the number of sentences was a continuous variable.

The study was granted an exemption from requiring ethics approval by the ethical review committee at Graduate School of Medicine, The University of Tokyo.

## Results

Inter-rater reliability was acceptable (ICC = 0.88, 95% CI = 0.80-0.93). The number of letters of the articles varied from 91 to 6,492. The contents of the articles included announcement of the date and time, location, cost of cancer screenings, recommendation to use screenings, and general information about cancer. The types of cancer that featured in the newsletters were mostly gastric, lung, colorectal, cervical, breast, and prostate cancer. Of the 129 articles that were evaluated, the total number of gain-framed sentences was 87, while that of loss-framed sentences was six (Table 2). The total number of gain-framed articles was 32 (24.8%) while that of loss-framed articles was zero (0%). Five articles (3.9%) were mixed-framed. Ninety-two articles (71.3%) were non-framed.

**Table 2:** **Frequency of framed messages**

Sentences				
	N	Mean value (S.D.)	Maximum	Minimum
Gain-framed	87	2.3 (1.82)	11	2
Loss-framed	6	1.2 (0.44)	2	1
**Articles**				
			**N**	**%**
Gain-framed article (a)			32	24.8
Loss-framed article (b)			0	0
Mixed-framed article (c)			5	3.9
Non-framed article (d)			92	71.3

(a) Conveying gain-framed and non-framed sentences.

(b) Conveying loss-framed and non-framed sentences.

(c) Conveying gain-framed, loss-framed, and non-framed sentences.

(d) Conveying only non-framed sentences, and no gain- or loss-framed sentences.

## Discussion

Based upon the framing postulate of Prospect Theory, the cancer screening announcement articles that we analyzed were not framed in the most effective way to motivate readers to obtain cancer screenings. The present study showed that most of the articles in municipal newsletters were non-framed (71.3%). These non-framed articles provided mainly factual information, such as the date and time, location, cost of cancer screenings, and the incidence of cancer. Message framing research has shown that gain- or loss- framed information is more persuasive than simple factual information [11, 25]. Thus, these non-framed articles may have been less likely to motivate readers to obtain cancer screenings.

According to previous studies [7, 11–14, 19], loss-framed cancer screening messages are more persuasive than gain-framed messages. However, in the present study, of the 129 articles, the total number of loss-framed sentences was only six, which was much fewer than that of gain-framed sentences (N = 87). Moreover, the number of loss-framed articles was zero (0%), while there were 32 (24.8%) gain-framed articles. Although five articles conveyed loss-framed sentences, these articles also conveyed gain-framed sentences (i.e., mixed-framed). Latimer et al. showed that mixed-framed content is less effective in persuasive communication than gain-framed content to motivate participation in physical activity [26]. (As mentioned above, gain-framed messages are considered to be more effective for promoting preventive behavior such as physical activity). Thus, mixed-framed content in the present study may be less persuasive than loss-framed content to motivate obtaining cancer screenings. The effectiveness of municipal newsletter articles in persuading people to have cancer screening might have been low.

Our findings are consistent with previous studies suggesting that the messages in health communication are not framed in the most effective way. Messages promoting preventive behavior, such as hand washing and cessation of smoking, could be more persuasive if gain-framed [7]. However, Jenner et al. showed that only 41% of the messages of hand hygiene posters were gain-framed and that some posters were mixed-framed [27]. Latimer et al. also showed that most of the smoking cessation print messages were non-framed, and that only 21.6% were gain-framed [20].

The small portion of loss-framed messages in the present analysis is consistent with the previous study that Wang-Buholzer et al. showed that public health campaigns adopted exclusively gain-framed messages [28]. The small portion of loss-framed messages in the present analysis may be due to the tendency of municipal newsletters to inform health content in a positive manner. Health promotion staff in public institutions tend to be reluctant to use loss-framed messages because they are anxious about that loss-framed messages can engender negative emotional reactions, such as fear. However, loss-framed messages arouse no greater fear than gain-framed messages or non-framed messages [17]. The persuasiveness of cancer screening announcement articles of municipal newsletters may be improved by adopting loss-framed messages as suggested by research.

We suggest three ways to increase loss-framed messages in cancer screening announcement articles. First, loss-framed messages could be increased in the headlines. In the present analysis, most of the headlines of small articles announcing merely date and location information were simply the name of the type of screening such as “Breast cancer screening”. Most of the other headlines were gain-framed, such as “Obtain cancer screening for early detection”. Loss-framed messages could be increased by inserting a loss-framed message or reframing gain-framed into loss-framed messages in the headlines; for example, a headline such as “Obtain cancer screening for early detection” could be reframed into a loss-framed headline (e.g., “Obtain cancer screening, if you don’t want to lose your health”).

Second, loss-framed messages could also be increased by supplementing non-framed messages with loss-framed information. An example of this possibility is that non-framed content, such as “One in 14 women suffer from breast cancer. They should have mammography regularly”, could be supplemented with loss-framed information (e.g., “One in 14 women suffer from breast cancer. If breast cancer is detected late, it can metastasize to other parts of your body, such as the lymph nodes, bones, lungs and liver, and can threaten your life. You should have mammography regularly”).

Third, the loss-framed message could be increased by reframing gain-framed messages into loss-framed messages. An example of this possibility is that a gain-framed message, such as “If cancer is detected and treated early, your physical, mental and economic burden is slight” could be rewritten in terms of losses (e.g., “If cancer is detected and treated late, your physical, mental, and economic burden is heavy”). Another example is as follows: “If a tumor is detected before malignant transformation, the operation is simple and the medical cost is small” could be reframed into “If a tumor is detected after malignant transformation, you need to have a major operation and pay a large medical cost.” To integrate message framing research into practice, the framing postulate of Prospect Theory needs to be widely known to the editors and writers of municipal newsletters and municipal health care staff.

The present study is the first study to analyze message framing of cancer screening information material. The main limitation of the present study is the descriptive design. Future studies need to assess the extent to which message framing of municipal newsletter articles affects readers’ attitude, intention, and behavior to obtain cancer screening. In addition, to what extent the present findings are generalizable to other cancer screening information materials is unclear. However, the present findings are important because the 23 Tokyo wards have a large population and strong political impact on other municipalities in Japan. Further, our findings are not unique to Japan, but are consistent with previous studies finding that public health services mainly use gain-framed messages [28].

## Conclusions

The number of loss-framed articles was zero in our study. In addition, there were fewer loss-framed messages used than gain-framed messages. Cancer screening announcement articles of municipal newsletters in Tokyo are not effectively framed to motivate people to obtain cancer screening. Most of the articles are non-framed or gain-framed. Loss-framed messages and articles should be increased to enhance the persuasiveness of the articles and motivate readers to obtain cancer screening.
